# Editorial: Molecular and cellular pathways underlying neurodegenerative disorders

**DOI:** 10.3389/fcell.2026.1943531

**Published:** 2026-07-31

**Authors:** Anastasia Bougea, Alberto Ouro, Allison B. Reiss

**Affiliations:** 1 First Department of Neurology, Medical School National and Kapodistrian University of Athens, Athens, Greece; 2 NeuroAging Group (NEURAL), Clinical Neurosciences Research Laboratory (LINC), Health Research Institute of Santiago de Compostela (IDIS), Santiago de Compostela, Spain; 3 Centro de Investigación Biomédica en Red en Enfermedades Neurodegenerativas (CIBERNED), Instituto de Salud Carlos III, Madrid, Spain; 4 Department of Foundations of Medicine, NYU Grossman Long Island School of Medicine, Mineola, NY, United States; 5 Department of Medicine, NYU Grossman Long Island School of Medicine, Mineola, NY, United States

**Keywords:** Alzheimer’s disease, autophagy, biomarkers, mitochondrial dysfunction, neurodegeneration, neuroinflammation, oxidative stress, Parkinson’s disease

Neurodegenerative disorders—chief among them Alzheimer’s disease (AD) and Parkinson’s disease (PD)—remain among the most intractable challenges in medicine. Decades of research organised around single, dominant hypotheses have yielded deep mechanistic insight but few disease-modifying therapies. The articles gathered in this Research Topic reflect a field in transition: away from linear, reductionist cascades and toward an integrative view in which neurodegeneration emerges from the interaction of multiple dysregulated molecular and cellular networks—mitochondrial dysfunction, oxidative stress, neuroinflammation, disrupted proteostasis, and programmed cell death—operating with striking cell-type and regional specificity. In assembling these seven contributions, our aim was to capture this shift across complementary levels of analysis: from upstream aetiological triggers to shared downstream effectors, and from mechanistic discovery to translational biomarkers and interventions.

Two contributions reframe how disease begins. Pluta and Ułamek-Kozioł synthesise extensive experimental and clinical evidence that cerebral ischaemia acts as an upstream driver of AD, dysregulating amyloid precursor protein, secretases, tau (MAPT), α-synuclein, apolipoproteins, and autophagy/apoptosis genes while compromising the blood–brain barrier. Their ischaemic model recasts amyloid and tau pathology as partly reactive to a vascular and energetic insult, illustrating how the classic “hypotheses” of AD may be interacting facets of one network rather than competing explanations. Complementing this conceptual re-examination, Ouro and colleagues review the therapeutic landscape of monoclonal antibodies and small molecules in AD, tracing the amyloid hypothesis under clinical scrutiny and the pivot toward tau-directed agents, kinase modulators, targeted protein degraders, and biomarker-guided, stage-specific precision medicine. Together they underscore a recurring lesson: robust target engagement does not guarantee clinical benefit when a single node is addressed in isolation.

A second pair brings high-resolution molecular detail to selective neuronal vulnerability in PD. Harbert reports a meta-analysis across seven human substantia nigra datasets showing that co-expression between *ALDH1A1*—a marker of vulnerable dopaminergic neurons and the rate-limiting enzyme in retinoic-acid synthesis—and core dopaminergic genes is selectively attenuated in PD, a signal that persists after adjusting for cell-type composition. The work links a retinoic-acid axis to the bulk-tissue transcriptomic signature of selective vulnerability. Extending this to spatial biochemistry, Xu and colleagues apply airflow-assisted desorption electrospray ionisation mass spectrometry imaging to map region-dependent metabolic disruption in MPTP-treated rats and the effects of nicotine, which broadly reversed the disease-associated metabolic trajectory by restoring dopaminergic metabolites, rebalancing GABA and serotonin, relieving mitochondrial and redox stress, and repairing glycerophospholipid metabolism. Their data reframe PD as a regionally heterogeneous yet mechanistically interconnected metabolic failure.

Convergence on shared effectors—apoptosis, mitochondrial quality control, oxidative stress, and autophagy—unites the interventional studies. Chen and colleagues synthesise evidence that exercise suppresses excessive neuronal apoptosis in PD by engaging three interconnected pathways: AMPK/Sirt1/PGC-1α (mitochondrial biogenesis), TLR/MyD88/NF-κB (neuroinflammation), and CaMKII/Beclin1/p62 (autophagy), with AMPK acting as a coordinating hub, while candidly appraising the translational limits of acute toxin-based rodent models. In a clinical counterpart, Krasnienkov and colleagues report a case series of adjunctive *Ginkgo biloba* extract (EGb 761) in PD, in which motor and cognitive gains accompanied shifts in mtDNA copy number, telomere length, and oxidative markers consistent with—though not proof of—mitophagy activation. Both illustrate how modifiable and adjunctive interventions may act on the very mitochondrial and inflammatory nodes implicated in disease initiation.

Finally, translational readouts anchor these mechanisms to accessible measures of brain health. Gill and colleagues use unbiased proteomics to identify a discovery blood-protein panel associated with accelerated brain-predicted-age discordance in neurologically healthy adults, implicating NF-κB, heat-shock protein, and Wnt signalling. The recurrence of NF-κB across this proteomic signature, the exercise literature, and AD therapeutics highlights neuroinflammation as a cross-disease axis and points toward minimally invasive detection of clinically silent, pre-pathological change—precisely when intervention may be most effective.

Read together, these seven articles articulate several unifying principles. First, heterogeneous upstream triggers—vascular, toxic, or age-related—feed into a shared set of downstream effectors, so that mitochondrial dysfunction, oxidative stress, neuroinflammation, and impaired proteostasis recur across AD and PD alike. Second, disease expression is governed by cell-type and regional vulnerability, demanding methods—single-nucleus deconvolution, spatial metabolomics—that resolve heterogeneity rather than average it away. Third, the persistent gap between preclinical mechanism and clinical benefit calls for combination and precision strategies, biomarker stratification, and honest appraisal of model limitations. We hope this Research Topic helps move the field from cataloguing individual pathways toward understanding their interactions—and, ultimately, toward interventions matched to the biology of the individual patient.

